# Temporal trends of low back pain burden and joinpoint and age-period-cohort analysis in China from 1990 to 2021

**DOI:** 10.1186/s12889-025-22774-5

**Published:** 2025-04-30

**Authors:** Bo Liang, Yue Wei, Heming Pei, Xiaoxuan Liang, Gong Chen, Lijun Pei

**Affiliations:** 1 Institute of Population Research, Peking University, No. 5 Yiheyuan Road, Haidian District, Beijing, 100871 China; 2 Department of Epidemiology, Columbia University Mailman School of Public Health, NY, USA

**Keywords:** Low back pain, Temporal trend, China, Global burden of disease, Age-period-cohort analysis

## Abstract

**Background:**

The burden of low back pain (LBP) is increasing rapidly. This study aimed to analyze the temporal trends of the LBP burden in China from 1990 to 2021.

**Methods:**

Data from the Global Burden of Disease (GBD) 2021 were used to examine the crude and age-standardized rates, along with their uncertainty interval (UI), for incidence, prevalence and disability adjusted life years (DALYs) per 100,000 for LBP, stratified by sex. The joinpoint regression model was applied to calculate the annual percent change (APC) and corresponding 95% confidence interval (*CI*) for the LBP burden. Additionally, an age-period-cohort analysis was conducted to assess the temporal trends in the LBP burden.

**Results:**

In 2021, LBP affected 100,093,745 individuals in China. The crude incidence, prevalence and DALYs rates were 3.05% (95% UI: 2.64–3.46%), 7.04% (95% UI: 6.12–7.94%) and 794.08 per 100,000 (95% UI: 557.48–1077.36), respectively. From 1990 to 2021, the age-standardized incidence, prevalence and DALYs rates declined annually by 0.71% (95% *CI*: 0.67–0.75%), 0.79% (95% *CI*: 0.75–0.83%), and 0.79% (95% *CI*: 0.75–0.84%), respectively. The LBP burden was higher in females than in males, with incidence rates rising with age. From the age of 45 onward, women exhibited significantly higher incidence rates than men. Over the past three decades, both period-specific and cohort-specific LBP incidence showed a downward trend.

**Conclusion:**

LBP remains a substantial public health burden in China, with variations across sex, age, period and cohort. Targeted healthcare policies and resource allocation should be prioritized for high-risk populations, particularly older adults and females.

**Supplementary Information:**

The online version contains supplementary material available at 10.1186/s12889-025-22774-5.

## Background

Low back pain (LBP), a prevalent musculoskeletal symptom, encompasses a range of symptoms from nonspecific discomfort and stiffness to severe pain. The pain is typically localized between the lower rib margin and the top of the buttock, sometimes accompanied by sciatica [1–2]. LBP is highly prevalent worldwide, affecting populations across all age groups and socioeconomic backgrounds in both developed and developing nations throughout life [3–4]. As the leading cause of years lived with disability (YLDs), LBP affected 619 million individuals globally in 2020, with projections exceeding 800 million by 2050 [5]. LBP restricts patients’ mobility, work capacity and quality of life, placing heavy economic and healthcare burden on individuals, families and nations [6]. Although LBP is not life-threatening, its significant contribution to YLDs makes it a major public health concern [7].

China, home to the world’s largest and fastest-growing aging population, faces an increasing burden of LBP, as the condition is highly prevalent among older adults [8]. Therefore, it is essential to analyze the long-term trends of LBP and its disease burden in China. While previous studies have explored the prevalence, YLDs, and risk factors associated with LBP using the Global Burden of Disease (GBD) 2019 data, the temporal trends and age-period-cohort dynamics remain unclear. This study utilized the most recent GBD 2021 data to examine the evolution of LBP burden from 1990 to 2021 in China, employing joinpoint regression and age-period-cohort analysis. The findings provide critical insights into the shifting patterns of LBP burden over the past three decades, including potential changes after the widespread of COVID-19.

## Methods

### Data source

A longitudinal trend analysis was performed to estimate the burden of LBP in China. All data, including crude and the age-standardized rates of prevalence, incidence, disability adjusted life years (DALYs, which measure both the years lost due to premature mortality and those lived with disability) were obtained from the GBD 2021 database, stratified by sex. The GBD 2021 study, conducted by the Institute for Health Metrics and Evaluation (IHME) at the University of Washington [9], provides a systematic scientific evaluation of the prevalence, incidence, and mortality rates of various diseases and injuries. It integrates publicly available data from 204 countries and territories, covering 371 diseases and injuries [10]. The GBD database applies age-standardization to crude rates of incidence, prevalence, YLDs, and DALYs using a standardized global reference population. Its goal is to offer standardized and comparable metrics for significant health concerns at the global level, aiming to enhance healthcare systems.

### Disease classification and burden indicators

LBP refers to chronic or acute pain occurring in the posterior torso, extending from the twelfth rib’s lower edge to the gluteal creases. This pain may be localized to this area or radiate to one or both lower limbs, which should last for a minimum of one day [8]. The International Classification of Diseases, 10th Revision (ICD-10), classifies LBP under codes M54.3 and M54.4 [4]. Given that LBP is an age-dependent condition and China’s population structure has undergone significant demographic shifts over the past three decades, age standardization is essential. Therefore, both crude and age-standardized indicators, including rates and uncertainty interval (UI) of incidence, prevalence and DALYs, were used in the study to assess the temporal trends in LBP burden from 1990 to 2021 in China.

### Statistical analysis

The joinpoint regression program (version 5.0.2) was used. This study calculated the age-standardized incidence rate (ASIR), age-standardized prevalence rate (ASPR) and age-standardized DALYs rate (ASDR) for total population and sex stratification. The joinpoint regression model was applied to estimate the annual percent change (APC) and its 95% confidence interval (*CI*). Moreover, to describe the overall trend of LBP from 1990 to 2021, the average annual percent change (AAPC) was calculated. AAPC provides a comprehensive summary by integrating segmented APCs, allowing for the comparison of change rates over time and the identification of long-term trends.

The age-period-cohort analysis was used to estimate the effect of age, period and cohort on LBP incidence in China. This method enhances the conventional descriptive approach by analyzing incidence rates across three dimensions. The age effect indicates the progression of aging, both societal and biological; the period effect captures shifts in LBP rates over time; and the cohort effect accounts for the unique risks and exposures that define specific birth cohorts [11]. This study mainly focused on the estimation of local drift, longitudinal age curve, rate ratios (RRs) for different periods and cohorts [12]. Incidence and population data were categorized into 5-year intervals from 1992 to 2021, covering individuals aged 30 to 84 years. This analysis was conducted using the online tool provided by the National Cancer Institute, USA, with statistical significance set at *P* < 0.05.

## Results

### LBP burden from 1990 to 2021 in China

#### Overall crude rates

**Fig. 1:**
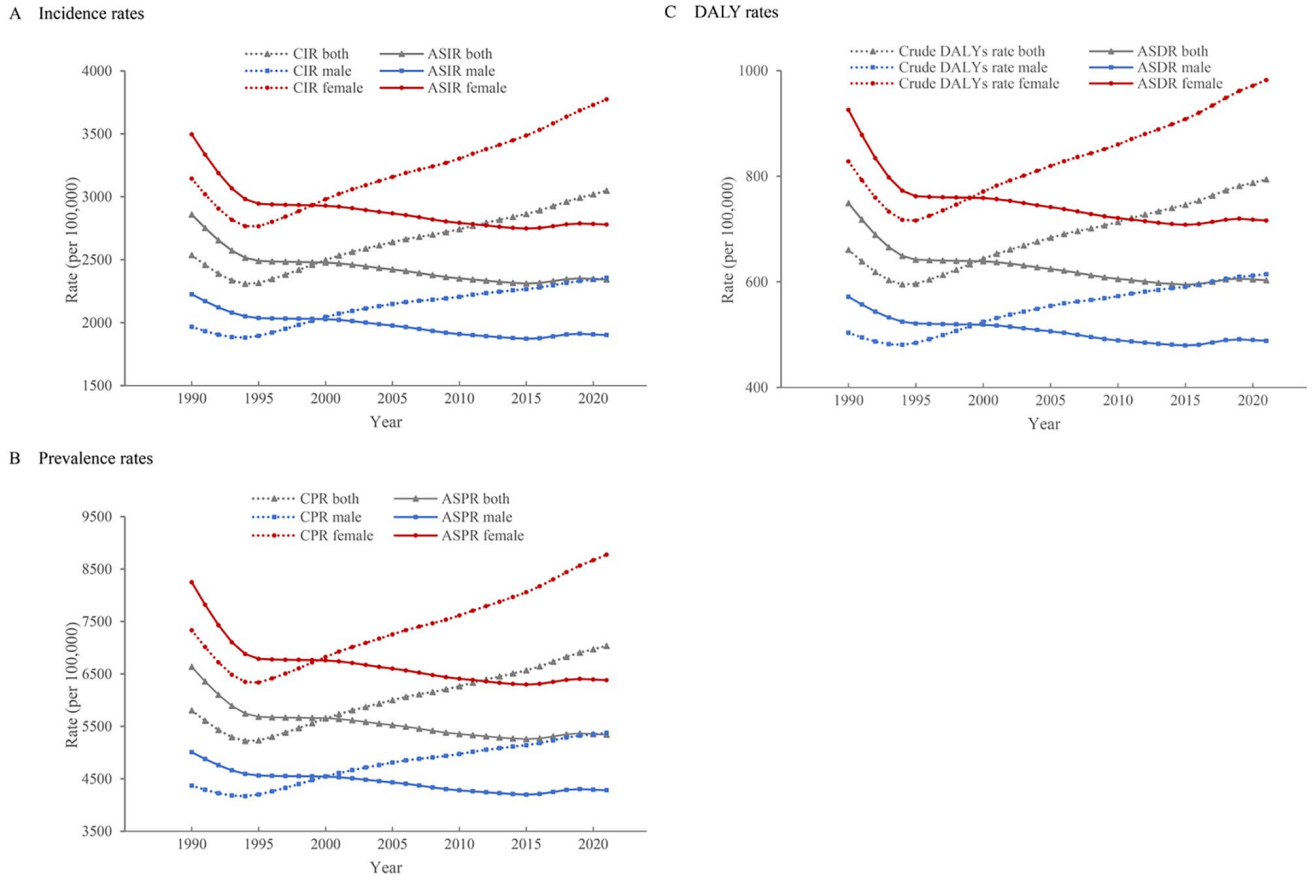
Trends of low back pain burden in China from 1990 to 2021, stratified by sex. Abbreviation: CIR, crude incidence rate; ASIR, age-standardized incidence rate; CPR, crude prevalence rate; ASPR, age-standardized prevalence rate; DALYs, disability adjusted life years; ASDR, age-standardized disability adjusted life years rate.

In 2021, an estimated 100,093,745 (95% UI: 87,128,172–113,014,315) individuals were suffering from LBP in China. Figure 1, Table S1 and S2 illustrate the trends in crude incidence rate (CIR), crude prevalence rate (CPR), crude DALYs rate, and ASIR, ASPR, and ASDR for LBP by sex from 1990 to 2021. Notably, substantial differences were observed between crude and age-standardized data. In 1990, the CIR, CPR and crude DALYs rate were 2.54% (95% UI: 2.22–2.89%), 5.80% (95% UI: 5.03–6.62%) and 660.71 per 100,000 (95% UI: 469.22–896.39), respectively. These increased to 3.05% (95% UI: 2.64–3.46%), 7.04% (95% UI: 6.12–7.94%) and 794.08 per 100,000 (95% UI: 557.48–1077.36) in 2021, respectively.

A distinct declining trend was observed from 1990 to 1994, marking a significant turning point, followed by a steady increase between 1994 and 2021. Specifically, between 1990 and 1994, the CIR, CPR and crude DALYs rate decreased by 8.99%, 10.03% and 9.92%, respectively. Subsequently, from 1994 to 2021, these rates increased by 32.05%, 34.73% and 33.43%, respectively.

#### Age-standardized rates

After age standardization, the ASIR, ASPR, and ASDR exhibited a distinct declining trend with minor fluctuations between 1990 and 2021. Specifically, the ASIR, and ASPR decreased from 2.86% (95% UI: 2.51–3.23%) and 6.64% (95% UI: 5.77–7.47%) in 1990 to 2.34% (95% UI: 2.06–2.64%) and 5.34% (95% UI: 4.66–5.98%) in 2021, respectively. Similarly, the ASDR declined from 749.03 (95% UI: 530.01–1013.84) per 100,000 in 1990 to 603.03 (95% UI: 427.63–810.16) per 100,000 in 2021 in China.

### Sex-specific trends in LBP burden

The trends in LBP burden for both sexes mirrored those of the general population. Overall, females had higher CIR, CPR, crude DALYs rates, ASIR, ASPR, and ASDR for LBP than males (Fig. 1, Table S1 and S2).

#### Male population

In 1990, the CIR, CPR and crude DALYs rate among males were 1.97% (95% UI: 1.71–2.25%), 4.37% (95% UI: 3.78–5.02%), and 503.53 per 100,000 (95% UI: 354.88–681.58), respectively. These rates decreased to 1.88% (95% UI: 1.65–2.16%), 4.17% (95% UI: 3.62–4.77%), and 480.89 per 100,000 (95% UI: 341.67–651.38), respectively, in 1994, before undergoing a notable increase to 2.36% (95% UI: 2.05–2.68%), 5.38% (95% UI: 4.65–6.09%) and 614.54 per 100,000 (95% UI: 433.53–833.32) in 2021, respectively. After age-standardization, the ASIR, ASPR, and ASDR in males exhibited an overall downward trend, decreasing by 14.55%, 14.48%, and 14.58%, respectively, between 1990 and 2021.

#### Female population

Among females, the CIR, CPR and crude DALYs rates showed a downward trend from 1990 to 1995, followed by a steady increase thereafter. In 1990, these rates were 3.14% (95% UI: 2.75–3.56%), 7.33% (95% UI: 6.36–8.34%), and 828.14 per 100,000 (95% UI: 590.00–1121.40), respectively. By 2020, they had increased to 3.77% (95% UI: 3.26–4.28%), 8.77% (95% UI: 7.62–9.86%), and 982.27 per 100,000 (95% UI: 687.52–1327.15), indicating a growing burden of LBP among females. After age-standardization, the ASIR, ASPR, and ASDR in females showed an overall decreasing trend, with reductions of 20.49%, 22.62%, and 22.65%, respectively, from 1990 to 2021.

[Figure 1 Trends of low back pain burden in China from 1990 to 2021, stratified by sex.]

### Joinpoint regression analysis of LBP disease burden in China

#### Trends in crude rates

Table 1 displays the joinpoint regression analysis for both crude and age-standardized rates of LBP from 1990 to 2021. Significant shifts in trend patterns were identified in both crude and age-standardized rates. The CIR exhibited an initial declining trend between 1990 and 1994, followed by a consistent increase until 2021, with an overall AAPC of 0.61 (95% *CI*: 0.58–0.64). A similar trend was observed in both males and females, with AAPCs of 0.60 (95% *CI*: 0.57–0.62) for males and 0.61 (95% *CI*: 0.58–0.64) for females. Similarly, both the CPR and crude DALYs rate declined between 1990 and 1994, followed by a significant rise from 1994 to 2021. The AAPCs for CPR were 0.69 (95% *CI*: 0.66–0.72) in males, 0.60 (95% *CI*: 0.57–0.64) in females, and 0.65 (95% *CI*: 0.61–0.68) for the overall population. Regarding crude DALYs rates, the AAPCs were 0.67 (95% *CI*: 0.64–0.70) for males, 0.58 (95% *CI*: 0.54–0.61) for females, and 0.61 (95% *CI*: 0.57–0.64) for the entire population.

**Table 1:** Log-transformed joinpoint trends of low back pain rates by sex in China

	Crude rates					Age-standardized rates			
	Trend/ Period	Incidence APC (95%CI)	Prevalence APC (95%CI)	DALYs APC (95%CI)		Trend/ Period	Incidence APC (95%CI)	Prevalence APC (95%CI)	DALYs APC (95%CI)
**Both**	1990–1994	-2.46*(-2.60;-2.32)	-2.75*(-2.91;-2.58)	-2.75*(-2.96;-2.53)		1990–1994	-3.23*(-3.37;-3.09)	-3.63*(-3.78;-3.48)	-3.61*(-3.75;-3.47)
	1994–2002	1.47*(1.41–1.54)	NA	1.49*(1.40–1.59)		1994–2001	-0.14*(-0.22;-0.07)	-0.14*(-0.21;-0.05)	-0.13*(-0.21;-0.05)
	1994–2003	NA	1.46*(1.40–1.52)	NA		2001–2014	-0.53*(-0.56;-0.50)	-0.56*(-0.59;-0.53)	-0.55*(-0.58;-0.52)
	2002–2015	0.84*(0.81–0.87)	NA	NA		2014–2019	0.37*(0.22–0.52)	0.43*(0.28–0.59)	0.41*(0.26–0.56)
	2002–2021	NA	NA	0.95*(0.93–0.98)		2019–2021	-0.15(-0.61-0.31)	-0.17(-0.65-0.32)	-0.22(-0.68-0.24)
	2003–2014	NA	0.91*(0.87–0.95)						
	2014–2021	NA	1.16*(1.09–1.23)						
	2015–2021	1.07*(0.99–1.15)							
AAPC (95%*CI*)	1990–2021	0.61*(0.58–0.64)	0.65*(0.61–0.68)	0.61*(0.57–0.64)		1990–2021	-0.63*(-0.67;-0.58)	-0.68*(-0.73;-0.64)	-0.68*(-0.73;-0.64)
**Male**	1990–1994	-1.17*(-1.34;-1.01)	-1.20*(-1.38;-1.02)	-1.20*(-1.37;-1.02)		1990–1994	-2.08*(-2.18;-1.98)	-2.19*(-2.29;-2.08)	-2.15*(-2.26;-2.04)
	1994–2002	1.50*(1.42–1.57)	NA	NA		1994–2001	-0.10*(-0.15;-0.05)	-0.10*(-0.15;-0.04)	NA
	1994–2003	NA	1.50*(1.43–1.56)	1.51*(1.45–1.57)		1994–2002	NA	NA	-0.15*(-0.19;-0.10)
	2002–2021	0.60*(0.58–0.61)	NA	NA		2001–2010	-0.65*(-0.68;-0.62)	-0.64*(-0.68;-0.61)	NA
	2003–2021		0.71*(0.69–0.73)	0.67*(0.65–0.69)		2002–2010	NA	NA	-0.67*(-0.72;-0.63)
						2010–2015	-0.43*(-0.53;-0.33)	-0.43*(-0.54;-0.33)	-0.42*(-0.53;-0.32)
						2015–2019	0.59*(0.43–0.74)	0.69*(0.52–0.86)	0.67*(0.50–0.84)
						2019–2021	-0.32*(-0.64;-0.01)	-0.32(-0.64-0.01)	-0.35*(-0.69;-0.01)
AAPC (95%*CI*)	1990–2021	0.60*(0.57–0.62)	0.69*(0.66–0.72)	0.67*(0.64–0.70)		1990–2021	-0.50*(-0.53;-0.46)	-0.49*(-0.53;-0.46)	-0.50*(-0.54;-0.46)
**Female**	1990–1994	-3.33*(-3.48;-3.19)	-3.74*(-3.91;-3.58)	-3.74*(-3.90;-3.58)		1990–1994	-4.00*(-4.18;-3.82)	-4.54*(-4.75;-4.34)	-4.54*(-4.75;-4.33)
	1994–2002	1.43*(1.37–1.50)	NA	NA		1994–2001	-0.16*(-0.26;-0.07)	-0.15*(-0.27;-0.04)	-0.16*(-0.27;-0.04)
	1994–2003	NA	1.40*(1.35–1.46)	1.40*(1.34–1.46)		2001–2014	-0.47*(-0.51;-0.44)	-0.53*(-0.57;-0.49)	-0.51*(-0.55;-0.47)
	2002–2014	0.98*(0.95–1.02)	NA	NA		2014–2021	0.23*(0.15–0.31)	0.28*(0.19–0.37)	0.24*(0.15–0.34)
	2003–2014	NA	1.03*(0.99–1.07)	1.03*(0.98–1.07)					
	2014–2021	1.34*(1.27–1.41)	1.45*(1.38–1.52)	1.33*(1.26–1.41)					
AAPC (95%*CI*)	1990–2021	0.61*(0.58–0.64)	0.60*(0.57–0.64)	0.58*(0.54–0.61)		1990–2021	-0.71*(-0.75;-0.67)	-0.79*(-0.83;-0.75)	-0.79*(-0.84;-0.75)

Abbreviation: DALYs, disability adjusted life years; APC, annual percent change; AAPC, average annual percent change; NA, not applicable. * means *P*<0.05.

#### Trends in age-standardized rates

After age-standardization, a notable shift in the trend of LBP disease burden was observed. Across the entire population, the ASIR, ASPR and ASDR demonstrated an overall declining trend, with AAPCs of -0.63 (95% *CI*: -0.67;-0.58) for ASIR, -0.68 (95% *CI*: -0.73;-0.64) for ASPR, and - 0.68 (95% *CI*: -0.73;-0.64) for ASDR. Specifically, these rates exhibited a downward trend from 1990 to 2014, followed by an increase between 2014 and 2019, and then remained stable thereafter. Among males, the ASIR and ASDR initially declined until 2015, followed by a rising trend between 2015 and 2019 and then a decreasing trend after 2019, with respective AAPCs of -0.50 (95% *CI*: -0.53;-0.46) and - 0.50 (95% *CI*: -0.54;-0.46). The ASPR of males exhibited a declining trend from 1990 to 2015, an upward trend between 2015 and 2019, and stabilized thereafter [AAPC=-0.49 (95% *CI*: -0.53;-0.46)]. For females, the ASIR, ASPR, and ASDR showed a declining trend from 1990 to 2014, followed by an increase after 2014, with AAPCs of -0.71 (95% CI: -0.75;-0.67) for ASIR, -0.79 (95% CI: -0.83;-0.75) for ASPR, and - 0.79 (95% CI: -0.84;-0.75) for ASDR.

[Table 1 Log-transformed joinpoint trends of low back pain rates by sex in China]

### Age-Period-Cohort model analysis of LBP incidence rates in China

#### Local drifts in LBP incidence

**Fig. 2:**
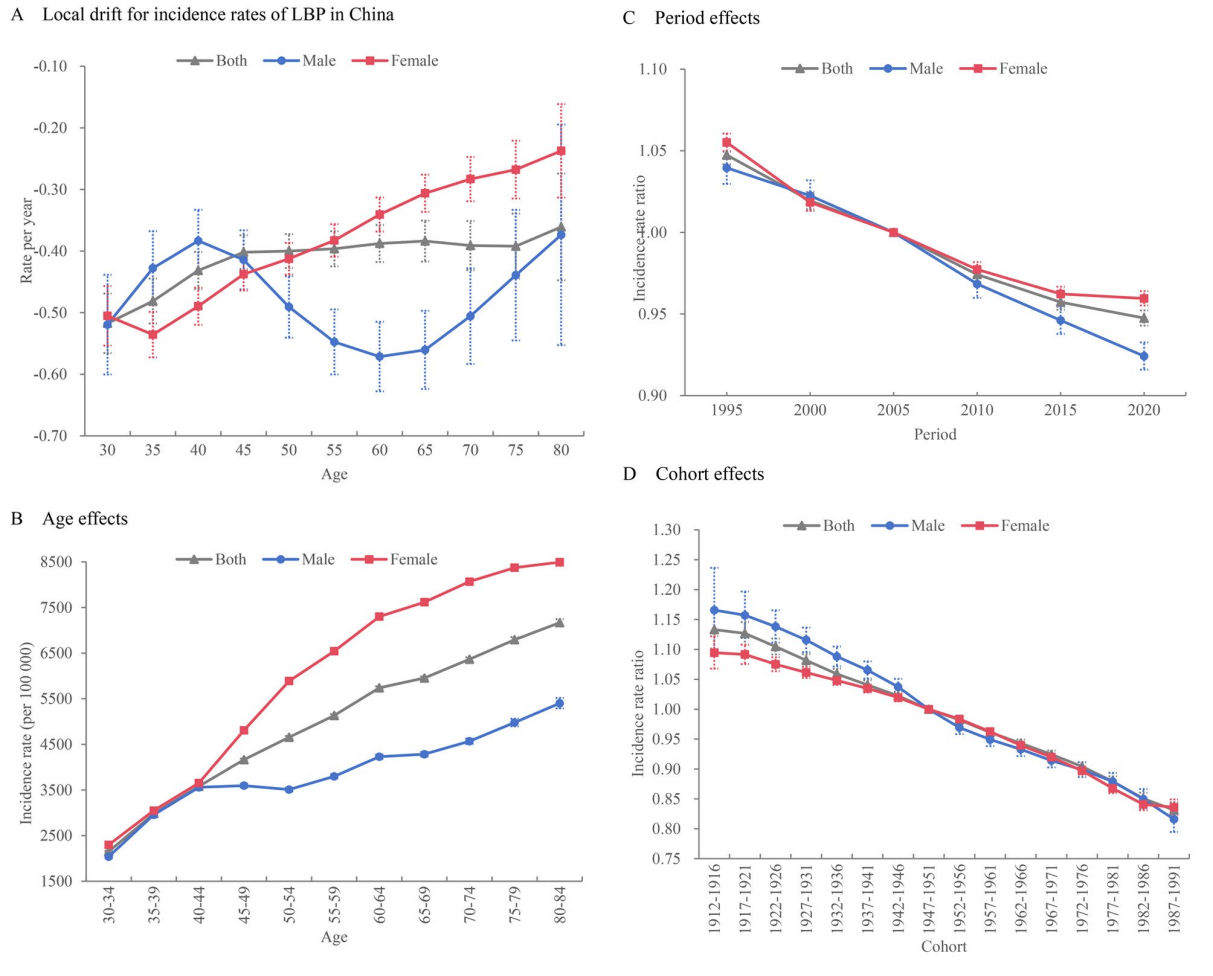
Age, period and cohort effects on low back pain (LBP) incidence in China. (**A**) Local drifts for incidence rates for LBP. (**B**) Longitudinal age curves for incidence rates of LBP. (**C**) Period rate ratio for incidence rates of LBP. (**D**) Cohort rate ratio for incidence rates of LBP

Figure 2(A) presents the local drifts from age-period-cohort analysis, estimating the AAPC in LBP incidence in China. Overall, the local drifts in LBP incidence increased with age, accompanied by fluctuations. These fluctuations were significantly greater in males compared to females. Specifically, for males, the local drifts of LBP incidence rose with age in the 30–44 and 65–84 age groups, but decreased with age in the 45–64 age group. Conversely, for females, the curve of local drifts displayed a J-shaped pattern across age groups, with the most notable decrease occurring before 40 years of age.

#### Longitudinal age curves of LBP incidence

Figure 2(B) displays the longitudinal age curves for LBP incidence for the entire population, males and females in China. The effect of age on LBP incidence showed a rising trend, peaking in the 80–84 age group. For males, LBP incidence sharply increased between the age of 30 and 44, remained stable between 45 and 54, and then increased more slowly after the age of 55. For females, LBP incidence rose more quickly with age among 40–64 years, with a slower increase after 65. Notably, from the age of 45, the incidence rate of LBP in females was significantly higher than in males of the same age.

#### Period and cohort effects on LBP incidence

Figure 2(C) and (D) illustrate the estimated effects of period and cohort on LBP incidence in China. The period rate ratios (RRs) for LBP incidence in China have shown a continuous and gradual decline from 1992 to 2021. The coefficients for the period effect have gradually diminished for both sexes. For males, the decline in incidence RRs was relatively consistent, whereas for females, the decline was faster between 1992 and 2001, but slowed after 2002. Similarly, the results for cohort effects generally showed a declining trend throughout the period. Furthermore, the cohort effect coefficients for both sexes exhibited a downward trend, with men experiencing a relatively steeper rate of decline. Notably, males had higher cohort effect coefficients than females before 1946. However, after that, the cohort effect factors for males converged with those of females. The APC model results for LBP prevalence and DALYs rates in China are shown in Fig.S1 and Fig.S2.

[Figure 2 Age, period and cohort effects on low back pain (LBP) incidence in China. (A) Local drifts for incidence rates for LBP. (B) Longitudinal age curves for incidence rates of LBP. (C) Period rate ratio for incidence rates of LBP. (D) Cohort rate ratio for incidence rates of LBP.]

## Discussion

This study aimed to assess the temporal trends in the incidence, prevalence and disease burden of LBP between 1990 and 2021, and estimate the age-period-cohort effects on LBP incidence rates in China utilizing data from GBD 2021 database. Globally, LBP has long been the leading cause of YLDs [4], a trend mirrored in China, placing a heavy burden on the nation. The findings provide valuable insights for policymakers to address LBP-related health challenges in China.

The CIR, CPR and crude DALYs rates for LBP in China exhibited an overall upward trend from 1990 to 2021, reaching 3.05% (95% UI: 2.64–3.46%), 7.04% (95% UI: 6.12–7.94%) and 794.08 (95% UI: 557.48–1077.36) per 100,000, respectively, in 2021. These rates initially declined between 1990 and 1994, before rising steadily thereafter. In particular, the increase in female rates after 1995 was notably steeper, likely reflecting demographic shifts such as population aging and increased life expectancy of females [13–17]. Aging is associated with musculoskeletal degeneration, including reduced muscle mass, intervertebral disc degeneration, and decreased bone density, all of which contribute to an increased risk of LBP [18–19]. After adjusting for age structure, a decrease in LBP burden was observed in China. The ASIR, ASPR and ASDR all showed declining trends with slight fluctuations between 1990 and 2021, with AAPC values of -0.71 (95% *CI*: -0.75;-0.67), -0.79 (95% *CI*: -0.83;-0.75), and - 0.79 (95% *CI*: -0.84;-0.75), respectively. This decline suggests improvements in overall health, quality of life, and health awareness among the younger generations, who engage in less physically strenuous work due to increased automation and mechanization [7–8, 20]. However, the underreporting or underestimation of LBP among younger individuals in China remains a concern, driven by high societal pressures and unhealthy working conditions prevalent in some industries, such as poor work design and excessive sedentary behavior [21–22]. Therefore, the study results warrant cautious interpretation.

There are substantial sex disparities in LBP burden in China. Females had higher crude and age-standardized incidence, prevalence and DALYs rates for LBP than males. Biological, psychological, and socialcultural factors contribute to these disparities. Biologically, hormonal fluctuations, pregnancy, and weight gain can strain the musculoskeletal system in females, increasing their susceptibility to LBP [23]. Psychologically, due to sex differences in pain sensitivity, females are more sensitive to pain, which increases the reporting and detection rates of LBP [24]. Socially, women often perform physical tasks that place excessive strain on their lower backs, such as household chores and caregiving [21]. Additionally, compared to men, women’s longer life expectancy may increase the risk and burden of LBP [22].

Age is a key demographic factor strongly correlated with LBP. The incidence rates rose steadily with age, peaking in the 80–84 age group. Several reasons may explain the age pattern. First, intervertebral disc degeneration, a natural aging process, is a crucial risk factor for LBP, thereby increasing the incidence rates in older adults [25]. Second, aging is often associated with pain. The occurrence of age-related pain could contribute to decreased physical activity, which in turn exacerbates pain and disability, creating a vicious cycle [8]. Third, poor sleep quality and insufficient rest, common in older adults, are risk factors for LBP that worsen with age [26–27]. Moreover, starting from the age of 45, women experienced significantly higher incidence rates of LBP compared to men of the same age. After women enter menopause, typically around age 45, estrogen levels decline, leading to a rise in osteoporosis incidence, which further increases the risk of LBP [28].

The period effects on LBP incidence rates in China showed a sustained and gradual decline from 1992 to 2021. Similarly, the cohort effects of LBP consistently declined among both men and women from the 1912–1916 to the 1987–1991 birth cohorts, indicating that individuals born in earlier cohorts had a greater risk than those born in later cohorts. The decline in period and cohort effects can be attributed to improvements in economic conditions, education, and healthcare access. Better nutrition, exercise, and healthcare in later generations likely reduced the overall risk of LBP [29].

The COVID-19 pandemic has introduced lifestyle shifts and public health measures, such as lockdowns, increased remote work and extended periods of sedentary behavior, which may have intensified LBP [30]. After 2019, a decline in the age-standardized burden of LBP occurred, potentially linked to changes in healthcare access and LBP management during the pandemic, such as delayed diagnoses. However, without specific data on pandemic-related behaviors and healthcare access, these conclusions remain speculative. Further research is needed to fully understand the pandemic’s impact on LBP and its long-term effects on musculoskeletal health.

This study provides updated estimates of the LBP burden by sex in China, with important public health implications. It highlights the need for targeted interventions, including improving ergonomics in workplaces, promoting physical activity, and enhancing health education to prevent and manage LBP, especially in vulnerable groups like women and older adults. Policymakers should prioritize LBP prevention and management as part of broader strategies to enhance musculoskeletal health in an aging population.

Several limitations should be acknowledged. First, the data came from GBD modeling, and the accuracy depended on the quality of information available in the country. This may result in slight differences from the actual situation, necessitating caution in interpreting the results. Second, while this study offers valuable national insights, its implications for individual-level health interventions are limited.

## Conclusion

This comprehensive analysis of the LBP burden in China from 1990 to 2021 reveals a declining trend in ASIR, ASPR, and ASDR. These improvements may be attributed to enhanced healthcare services, increased public awareness, and better preventive measures. Despite this progress, China continues to face a substantial LBP burden, with significant disparities observed across sex, age, period, and cohort. Comprehensive strategies to prevent and manage LBP are crucial, with particular emphasis on vulnerable populations, such as older adults and females. Efforts should prioritize promoting regular physical activity and targeted exercise programs to strengthen back muscles and improve flexibility. Improving workplace ergonomics and enhancing health education are also vital to reducing LBP risk. Moreover, integrating LBP and other musculoskeletal disorders prevention and management into primary healthcare services is essential to ensure early detection and timely intervention.

## Electronic supplementary material

Below is the link to the electronic supplementary material.

Supplementary Material 1

## Data Availability

The data are available in the Global Burden of Disease (GBD) database repository, (https://vizhub.healthdata.org/gbd-results/).

## Abbreviations

LBP: Low back pain
YLDs: Years lived with disability
GBD: Global Burden of Disease
DALYs: Disability adjusted life years
IHME: The Institute for Health Metrics and Evaluation
ICD-10: The International Classification of Diseases, 10th Version
UI: Uncertainty interval
ASIR: Age-standardized incidence rate
ASPR: Age-standardized prevalence rate
ASDR: Age-standardized DALYs rate
APC: Annual percent change
AAPC: Average annual percent change
CI: Confidence interval
RR: Rate ratio
CIR: Crude incidence rate
CPR: Crude prevalence rate
