# Supplementary material for: Temporal trends of low back pain burden and joinpoint and age-period-cohort analysis in China from 1990 to 2021
Source: BMC Public Health. 2025 Apr 30;25:1598. doi: 10.1186/s12889-025-22774-5 (PMC12042390; doi:10.1186/s12889-025-22774-5)
Supplement: Supplementary file 1 — Supplementary Material 1 [file 12889_2025_22774_MOESM1_ESM.docx]

**Supplementary Material**

Table S1. Crude rates of low back pain in China from 1990 to 2021, stratified by gender (per 100,000)

|  | Crude Incidence Rate | | |  | Crude Prevalence Rate | | |  | Crude DALY Rate | | |
| --- | --- | --- | --- | --- | --- | --- | --- | --- | --- | --- | --- |
| Year | Both | Male | Female |  | Both | Male | Female |  | Both | Male | Female |
| 1990 | 2536.77 | 1966.80 | 3143.97 |  | 5803.95 | 4369.96 | 7331.64 |  | 660.71 | 503.54 | 828.14 |
| 1991 | 2458.39 | 1932.58 | 3019.62 |  | 5608.08 | 4291.28 | 7013.58 |  | 638.73 | 494.62 | 792.56 |
| 1992 | 2388.58 | 1904.11 | 2906.45 |  | 5431.27 | 4224.82 | 6720.94 |  | 618.64 | 486.91 | 759.45 |
| 1993 | 2335.22 | 1885.47 | 2816.46 |  | 5293.61 | 4180.39 | 6484.80 |  | 603.21 | 481.95 | 732.95 |
| 1994 | 2308.82 | 1881.35 | 2766.43 |  | 5221.81 | 4170.69 | 6347.05 |  | 595.14 | 480.89 | 717.43 |
| 1995 | 2315.56 | 1895.14 | 2765.53 |  | 5232.19 | 4201.21 | 6335.68 |  | 596.31 | 484.45 | 716.03 |
| 1996 | 2346.23 | 1921.35 | 2800.65 |  | 5301.81 | 4261.25 | 6414.68 |  | 604.31 | 491.38 | 725.08 |
| 1997 | 2381.26 | 1950.71 | 2841.17 |  | 5381.13 | 4328.10 | 6505.95 |  | 613.22 | 499.04 | 735.19 |
| 1998 | 2419.21 | 1982.22 | 2885.26 |  | 5467.12 | 4399.88 | 6605.31 |  | 622.85 | 507.13 | 746.27 |
| 1999 | 2459.69 | 2014.20 | 2933.95 |  | 5560.88 | 4474.18 | 6717.79 |  | 633.43 | 515.70 | 758.76 |
| 2000 | 2498.31 | 2044.94 | 2980.07 |  | 5650.99 | 4546.77 | 6824.35 |  | 643.79 | 524.11 | 770.96 |
| 2001 | 2533.17 | 2071.39 | 3022.97 |  | 5734.21 | 4611.04 | 6925.54 |  | 653.12 | 531.43 | 782.19 |
| 2002 | 2562.61 | 2093.30 | 3059.50 |  | 5805.77 | 4666.11 | 7012.41 |  | 661.36 | 537.83 | 792.14 |
| 2003 | 2588.75 | 2112.44 | 3092.13 |  | 5870.84 | 4716.02 | 7091.28 |  | 668.79 | 543.64 | 801.06 |
| 2004 | 2614.54 | 2130.67 | 3124.95 |  | 5935.95 | 4763.49 | 7172.71 |  | 676.13 | 548.99 | 810.26 |
| 2005 | 2639.63 | 2147.91 | 3157.35 |  | 6000.59 | 4810.09 | 7254.08 |  | 683.55 | 554.41 | 819.51 |
| 2006 | 2663.36 | 2163.25 | 3189.03 |  | 6062.13 | 4851.50 | 7334.64 |  | 690.59 | 559.28 | 828.62 |
| 2007 | 2682.36 | 2173.95 | 3215.94 |  | 6112.31 | 4882.57 | 7402.94 |  | 696.27 | 562.70 | 836.45 |
| 2008 | 2699.14 | 2182.21 | 3240.90 |  | 6156.59 | 4908.34 | 7464.83 |  | 701.26 | 565.55 | 843.50 |
| 2009 | 2718.49 | 2192.17 | 3269.40 |  | 6205.94 | 4937.53 | 7533.61 |  | 706.92 | 568.85 | 851.45 |
| 2010 | 2742.06 | 2205.27 | 3303.24 |  | 6264.77 | 4973.73 | 7614.46 |  | 713.44 | 572.89 | 860.38 |
| 2011 | 2769.58 | 2221.49 | 3341.93 |  | 6332.69 | 5016.95 | 7706.67 |  | 721.00 | 577.71 | 870.62 |
| 2012 | 2794.18 | 2234.92 | 3377.72 |  | 6393.98 | 5053.77 | 7792.40 |  | 727.55 | 581.56 | 879.88 |
| 2013 | 2816.78 | 2245.93 | 3412.19 |  | 6450.68 | 5084.69 | 7875.42 |  | 733.55 | 584.70 | 888.80 |
| 2014 | 2840.10 | 2256.60 | 3448.69 |  | 6510.00 | 5115.11 | 7964.87 |  | 739.90 | 587.95 | 898.38 |
| 2015 | 2863.21 | 2265.86 | 3486.52 |  | 6568.19 | 5141.21 | 8057.16 |  | 746.07 | 590.65 | 908.23 |
| 2016 | 2891.65 | 2279.11 | 3531.29 |  | 6642.88 | 5180.27 | 8170.19 |  | 753.91 | 594.74 | 920.12 |
| 2017 | 2925.71 | 2297.31 | 3582.52 |  | 6734.07 | 5233.75 | 8302.24 |  | 763.52 | 600.36 | 934.06 |
| 2018 | 2960.67 | 2315.75 | 3635.42 |  | 6826.63 | 5285.81 | 8438.70 |  | 773.38 | 605.90 | 948.59 |
| 2019 | 2992.72 | 2331.09 | 3685.54 |  | 6906.91 | 5323.83 | 8564.60 |  | 781.66 | 609.69 | 961.74 |
| 2020 | 3018.92 | 2340.99 | 3729.24 |  | 6968.82 | 5347.38 | 8667.74 |  | 787.43 | 611.72 | 971.54 |
| 2021 | 3048.68 | 2356.54 | 3774.16 |  | 7035.25 | 5376.78 | 8773.61 |  | 794.08 | 614.54 | 982.27 |

Table S2. Age-standardized rates of low back pain in China from 1990 to 2021, stratified by gender (per 100,000)

|  | Age-standardized Incidence Rate | | |  | Age-standardized Prevalence Rate | | |  | Age-standardized DALY Rate | | |
| --- | --- | --- | --- | --- | --- | --- | --- | --- | --- | --- | --- |
| Year | Both | Male | Female |  | Both | Male | Female |  | Both | Male | Female |
| 1990 | 2859.38 | 2225.35 | 3495.28 |  | 6635.49 | 5007.60 | 8247.28 |  | 749.03 | 571.73 | 925.83 |
| 1991 | 2752.18 | 2170.86 | 3334.91 |  | 6357.44 | 4878.71 | 7820.13 |  | 717.92 | 557.11 | 878.15 |
| 1992 | 2653.98 | 2120.98 | 3187.86 |  | 6102.68 | 4760.76 | 7428.38 |  | 689.13 | 543.51 | 834.05 |
| 1993 | 2572.51 | 2079.52 | 3066.03 |  | 5891.74 | 4662.92 | 7104.41 |  | 665.51 | 532.45 | 797.79 |
| 1994 | 2515.55 | 2050.22 | 2981.20 |  | 5745.10 | 4594.07 | 6880.22 |  | 649.03 | 524.61 | 772.64 |
| 1995 | 2490.85 | 2036.93 | 2945.17 |  | 5683.04 | 4563.23 | 6787.86 |  | 642.05 | 521.13 | 762.22 |
| 1996 | 2486.24 | 2033.88 | 2939.20 |  | 5673.33 | 4556.59 | 6776.16 |  | 641.05 | 520.39 | 761.06 |
| 1997 | 2483.68 | 2032.25 | 2935.89 |  | 5668.42 | 4553.13 | 6770.63 |  | 640.43 | 519.96 | 760.30 |
| 1998 | 2482.03 | 2031.22 | 2933.89 |  | 5665.60 | 4551.00 | 6767.99 |  | 639.97 | 519.54 | 759.89 |
| 1999 | 2480.29 | 2029.97 | 2931.88 |  | 5662.48 | 4548.35 | 6764.85 |  | 639.54 | 519.25 | 759.36 |
| 2000 | 2477.60 | 2027.73 | 2928.64 |  | 5656.91 | 4543.52 | 6758.15 |  | 639.05 | 518.76 | 758.82 |
| 2001 | 2471.20 | 2022.00 | 2921.21 |  | 5642.16 | 4531.00 | 6740.15 |  | 637.33 | 517.31 | 756.70 |
| 2002 | 2460.42 | 2012.18 | 2909.13 |  | 5616.51 | 4509.44 | 6709.48 |  | 634.58 | 514.96 | 753.45 |
| 2003 | 2447.35 | 2000.18 | 2894.65 |  | 5585.07 | 4483.09 | 6672.07 |  | 631.13 | 512.05 | 749.35 |
| 2004 | 2434.11 | 1987.85 | 2880.03 |  | 5553.08 | 4456.04 | 6633.95 |  | 627.52 | 508.91 | 745.14 |
| 2005 | 2422.67 | 1976.96 | 2867.64 |  | 5525.44 | 4432.28 | 6601.30 |  | 624.51 | 506.31 | 741.58 |
| 2006 | 2410.25 | 1965.11 | 2854.36 |  | 5495.46 | 4406.06 | 6566.85 |  | 621.25 | 503.46 | 737.83 |
| 2007 | 2394.35 | 1950.06 | 2837.50 |  | 5457.29 | 4372.42 | 6524.00 |  | 617.04 | 499.57 | 733.26 |
| 2008 | 2377.54 | 1934.14 | 2819.76 |  | 5416.98 | 4336.76 | 6479.14 |  | 612.54 | 495.46 | 728.37 |
| 2009 | 2362.40 | 1919.71 | 2803.81 |  | 5380.62 | 4304.50 | 6438.60 |  | 608.59 | 491.81 | 724.10 |
| 2010 | 2351.54 | 1909.16 | 2792.48 |  | 5354.31 | 4281.05 | 6409.18 |  | 605.61 | 489.10 | 720.80 |
| 2011 | 2342.94 | 1901.07 | 2783.03 |  | 5333.25 | 4263.08 | 6384.51 |  | 603.30 | 487.08 | 718.12 |
| 2012 | 2333.48 | 1892.75 | 2772.10 |  | 5310.28 | 4244.40 | 6356.85 |  | 600.60 | 484.80 | 714.94 |
| 2013 | 2324.30 | 1884.77 | 2761.43 |  | 5288.21 | 4226.49 | 6330.40 |  | 598.02 | 482.63 | 711.92 |
| 2014 | 2316.48 | 1877.73 | 2752.73 |  | 5269.75 | 4210.87 | 6309.25 |  | 595.95 | 480.91 | 709.52 |
| 2015 | 2311.26 | 1872.22 | 2747.81 |  | 5257.98 | 4199.09 | 6297.78 |  | 594.57 | 479.51 | 708.19 |
| 2016 | 2315.79 | 1876.29 | 2752.57 |  | 5270.93 | 4211.20 | 6311.30 |  | 595.96 | 480.88 | 709.57 |
| 2017 | 2330.24 | 1891.02 | 2766.27 |  | 5308.93 | 4249.58 | 6348.07 |  | 600.08 | 485.09 | 713.49 |
| 2018 | 2345.39 | 1906.31 | 2780.75 |  | 5348.46 | 4289.15 | 6386.69 |  | 604.52 | 489.59 | 717.79 |
| 2019 | 2351.89 | 1912.11 | 2787.80 |  | 5365.76 | 4304.87 | 6405.61 |  | 606.35 | 491.24 | 719.80 |
| 2020 | 2347.27 | 1906.50 | 2784.11 |  | 5354.01 | 4293.46 | 6394.05 |  | 604.56 | 489.75 | 717.78 |
| 2021 | 2342.46 | 1901.62 | 2779.16 |  | 5342.10 | 4282.30 | 6381.38 |  | 603.03 | 488.36 | 716.15 |

A Local drift for prevalence rates of LBP in China

B Age effects

C Period effects

D Cohort effects

Figure S1. Age, period and cohort effects on low back pain (LBP) prevalence in China. (A) Local drifts for prevalence rates for LBP. (B) Longitudinal age curves for prevalence rates of LBP. (C) Period rate ratio for prevalence rates of LBP. (D) Cohort rate ratio for prevalence rates of LBP.

A Local drift for DALY rates of LBP in China

B Age effects

C Period effects

D Cohort effects

Figure S2. Age, period and cohort effects on low back pain (LBP) DALY rates in China. (A) Local drifts for DALY rates for LBP. (B) Longitudinal age curves for DALY rates of LBP. (C) Period rate ratio for DALY rates of LBP. (D) Cohort rate ratio for DALY rates of LBP.

Abbreviation: DALY, disability adjusted life years.

**Explanation of some terms used in the study**

1. Jointpoint Regression Models

Jointpoint regression models are a set of statistical tools used to analyze trends in data over time. They are particularly useful for identifying points at which the trend changes significantly, known as "joinpoints". This technique is designed to detect years where there are statistically significant abrupt changes in temporal trends. The models calculate the average annual percentage change (AAPC) and annual percentage change (APC), which help in understanding whether the trend is increasing or decreasing over the specified period.

1. Age-Period-Cohort (APC) Analysis

Age-Period-Cohort analysis is a method used to decompose trends in health outcomes into the effects of age, period, and cohort. This analysis helps in understanding how these three factors contribute to the observed trends. The APC model reflects a disease’s incidence or mortality time trend by age, period, and cohort. It's a technique that allows researchers to estimate the distinct effects of each component, despite the inherent linear associations among age, period, and cohort.

Key Terms:

Local Drift: This refers to the logarithmic linear trend of age standardized rates for each age group after adjusting for period and cohort effects. It helps in understanding the specific trends within each age group.

Age Effect: This represents the impact of age on a certain outcome as it increases。It is one of the most important determinants for disease occurrence. In the context of APC analysis, age effects are considered to reflect the natural course of disease with age.

Period Effect: This term indicates the independent effect of the current period after adjusting for age and cohort effects. It reflects the influence of time-specific factors on the outcome.

Cohort Effect: This represents the cumulative effect of age, period, and cohort on disease risk. It captures the influence of birth cohort-specific factors on the outcome.
